# Dual Burden of Kidney Disease and Cognitive Impairment in the Longitudinal Aging Study in India-Diagnostic Assessment of Dementia-Cohort

**DOI:** 10.1016/j.ekir.2026.106688

**Published:** 2026-07-01

**Authors:** Aradhana Nayak, Sandhya G., Vamsi K. Yenamandra, Jonas S. Sundarakumar, Latha Diwakar, Thomas Gregor Issac, Swaminathan Sundararaman

**Affiliations:** 1 All India Institute of Medical Sciences, Bhubaneswar, Odisha, India; 2 Centre for Brain Research, Indian Institute of Science, Bengaluru, Karnataka, India; 3 Department of Nephrology, Tata-IISc Medical School, Indian Institute of Science, Bengaluru, Karnataka, India

**Keywords:** chronic kidney disease, cognitive impairment, creatinine to cystatin C ratio, LASI-DAD, kidney-brain axis

## Abstract

**Introduction:**

Chronic kidney disease (CKD) and cognitive impairment are being increasingly recognized as major public health challenges in aging populations, yet their interrelationship remains underexplored in India.

**Methods:**

Data from Longitudinal Aging Study in India- Diagnostic Assessment of Dementia (LASI-DAD) cohort was analyzed. Estimated glomerular filtration rate (eGFR) was estimated using serum creatinine, cystatin C, and combined CKD-Epidemiology Collaboration (CKD-EPI) equations. Cognitive performance was assessed using Hindi Mental State Examination (HMSE) and domain-specific cognitive tests.

**Results:**

The prevalence of eGFR <60 ml/min per 1.73 m^2^ was 22.66% using CKD-EPI creatinine and cystatin C (eGFRcr-cys) equation representing one of the highest reported estimates among community-dwelling older adults. Reduced eGFR showed null associations with HMSE scores and performance in cognitive domains. Risk of cognitive impairment was similar in those with eGFR ≥ 60 ml/min per 1.73 m^2^ when compared with eGFR < 60 ml/min per 1.73 m^2^. However, the creatinine to cystatin C ratio demonstrated a positive association (ρ = 0.18, *P* value: < 0.001) with cognitive performance.

**Conclusion:**

No significant association between eGFR and cognition was found in the LASI-DAD cohort. High prevalence of reduced eGFR and cognitive impairment may be attributed to age, modifiable risk factors, and region-specific differences, highlighting the need for further longitudinal research in low- and middle-income countries (LMICs).

## Graphical abstract

**Figure:**
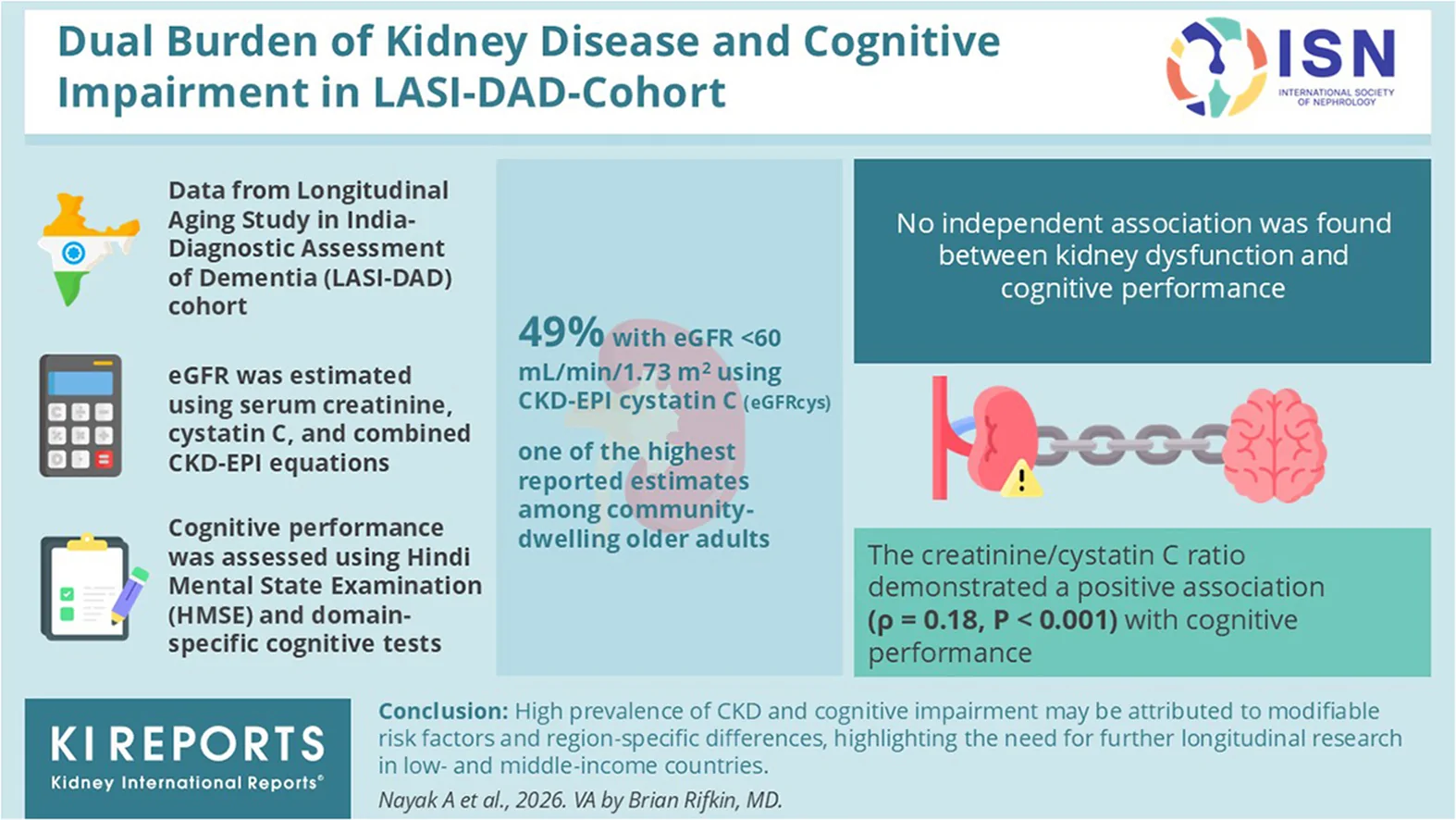

The worldwide prevalence of dementia was 55 million in 2020, with 60% of these individuals living in LMICs.^1^^,^^2^ In India, the prevalence of dementia is estimated to be 7.4% in adults aged ≥ 60, affecting about 8.8 million people.^3^ As the global population ages, the burden of dementia is expected to increase. According to the *Lancet* standing commission on dementia prevention, intervention, and care (2024), up to 45% of dementia cases could be prevented or delayed by addressing modifiable risk factors^4^ and highlighting the importance of early risk identification for dementia and mild cognitive impairment. These modifiable risk factors have a greater impact on dementia in LMICs than in high-income countries.^5^

CKD is recognized as a major contributor to cardiovascular risk and numerous recent studies have established a strong link between CKD and cognitive impairment,^6^, ^7^, ^8^, ^9^ mostly among Caucasian populations. The shared microvascular structure of the brain and kidneys makes both organs vulnerable to hemodynamic stress and systemic damage.^10^ Although associated cardiovascular disease may act as the primary link^11^^,^^12^, other studies point to the role of uremic toxins that can damage the brain’s monoaminergic system, neural stem cells, or glymphatic system.^13^ Impaired amyloid-β clearance from kidney dysfunction may also increase the risk.^14^^,^^15^

A key gap in this field is the paucity of data from LMICs, as most evidence derives from high-income settings and may not be generalizable because of regional differences in genetics, environment, and CKD etiology.^16^ Despite India being one of the countries with the highest global CKD burden,^17^ research linking kidney dysfunction to cognitive decline in the Indian population remains scarce.^18^ This relationship remains largely unexplored in older adults, a vulnerable and rapidly growing population, underscoring the need to identify at-risk individuals to guide prevention and reduce dementia burden.

Creatinine levels are associated with muscle mass whereas cystatin C levels are associated with visceral fat.^19^^,^^20^ In elderly populations, ratio of creatinine to cystatin C (sarcopenia index) reflects reduced muscle mass and physiological reserve.^21^ This ratio is particularly relevant in the South-East Asian population, who have a higher propensity to store visceral fat compared with the Caucasian population.^22^ A lower ratio is associated with poorer cognitive performance, higher risk of mild cognitive impairment and Alzheimer’s disease, and increased mortality.^23^, ^24^, ^25^, ^26^

The LASI-DAD is India’s first nationally representative study on cognitive aging and dementia.^20^ Our study investigates the relationship between renal dysfunction and cognitive functioning among older adults, using data from the LASI-DAD cohort. Specifically, we aim to— (i) examine the association between eGFR and cognitive performance in older Indian adults and (ii) explore the potential of the creatinine to cystatin C ratio as a biomarker for cognitive functioning in this older Indian population.

## Methods

### Data Source

The LASI is a longitudinal study of > 73,000 individuals aged ≥ 45 in India.^27^ The Wave 1 of LASI-DAD drew a subsample of respondents from the baseline LASI sample aged ≥ 60 from 18 states and union territories. A 2-stage stratified random sampling approach was adopted, oversampling individuals at high risk of cognitive impairment to ensure enough respondents with dementia or mild cognitive impairment.

Data from 4096 participants of LASI-DAD Wave 1 was taken for this cross-sectional study. Complete eGFR cystatin C (eGFRcys) data was present for *N* = 2604; eGFR creatinine (eGFRcr) data for *N* = 2859; eGFRcr-cys data for *N* = 2604; and subjects with missing data were excluded from specific statistical analyses.

### Kidney Function Parameters

Serum creatinine was measured by Jaffe’s method using *Architect ci8200* platform (Abbott Laboratories, Abbott Park, IL). Serum cystatin C was measured by polystyrene-enhanced immunoturbidimetric assay using BNProSpec (Siemens Healthcare Diagnostics, Tarrytown, NY). eGFR was calculated by 3 formulae, as follows: eGFRcys: Chronic Kidney Disease Epidemiology Collaboration (CKD-EPI) cystatin C equation (2012), eGFRcr: CKD-EPI creatinine equation (2021), and eGFRcr-cys: CKD-EPI creatinine-cystatin equation (2021).^28^

Reduced eGFR was defined as eGFR < 60ml/min per 1.73 m^2^ based on a single baseline measurement.

### Cognitive Measures

The HMSE score was used as a measure of global cognition. It is a culturally adapted version of the Mini-Mental State Examination designed to assess cognitive function in Hindi-speaking and low-literacy populations.^29^

HMSE ≤ 23 was defined as cognitive impairment. Domain-specific cognitive performance (orientation, executive functioning, language/fluency, memory, and visuospatial domains) was assessed using summary variables, based on the factor structure of the LASI-DAD cognitive battery described by Gross *et al.*^30^

### Covariates

Socioeconomic and demographic variables such as age, sex, years of education, and the following health-related variables: obesity, low-density lipoprotein levels, diabetic status, smoking status, alcohol status, hypertension, Center for Epidemiologic Studies Depression Scale score, and previously diagnosed cardiovascular event history were considered in the analysis. Conceptually relevant covariates were included based on previous literature on known risk factors for cognitive impairment.

### Statistical Analyses

First, we ran descriptive analyses to report the prevalence of kidney disease (based on eGFR) in the cohort. We used Kolmogorov-Smirnov Test for normality testing. Multivariable regression analysis (logistic and linear) were used to analyze the relationship of eGFR with cognitive scores. For all linear regression analyses, model 1 was unadjusted, model 2 was adjusted for age and sex, model 3 was additionally adjusted for years of education, obesity, low-density lipoprotein cholesterol, diabetes, smoking, alcohol consumption, hypertension, Center for Epidemiologic Studies Depression Scale score, and previously diagnosed cardiovascular event history. Spearman correlation was used to correlate creatinine to cystatin C ratio with HMSE and domain-wise cognitive scores. After excluding subjects with missing data for kidney parameters (eGFR) and other covariates, 2345 subjects were included in the regression analysis. Statistical analyses were performed using Python software (version 3.12; Python Software Foundation, Beaverton, OR).

## Results

### Demographics

The LASI-DAD Wave 1 participants had a median age of 68 years, 46.12% were males and 53.88% were females. The median eGFRcys, eGFRcr, and eGFRcr-cys levels were 60.38, 89.98, and 75.00 ml/min per 1.73 m^2^, respectively. The characteristics of all the participants of the LASI-DAD cohort compared with the subsample of LASI-DAD participants in our study (with eGFR data available) are depicted in Table 1. Based on different methods of eGFR calculation, distribution of eGFR levels among the sample is depicted in Table 2.

**Table 1:** This table depicts the gender distribution of subjects, median value of age, education, eGFR, and HMSE score and percentage prevalence of comorbidities in the LASI-DAD Wave 1 sample compared with the samples with eGFRcys, eGFRcr, and eGFRcr-cys data present respectively

Characteristics	All participants (*N* = 4096)	eGFRcys samples present (*n* = 2604)	eGFRcr samples present (*n* = 2859)	eGFRcr-cys samples present (*n* = 2604)	Kruskal Wallis H statistic, (*P* value)
Median value (IQR)					
Age (yrs)	68.00 (64.00–74.00)	68.00 (64.00–74.00)	68.00 (64.00–74.00)	68.00 (64.00–74.00)	0.58 (0.75)
Yrs of education	0.00 (0.00–7.00)	1.00 (0.00–7.00)	1.00 (0.00–7.00)	1.00 (0.00–7.00)	0.63 (0.73)
eGFRcys	#	60.38 (47.13–74.32)	60.64 (47.62–74.59)	60.38 (47.13–74.32)	0.67 (0.71)
eGFRcr	#	89.59 (77.09–96.09)	89.98 (77.47–96.40)	89.59 (77.09–96.09)	1.88 (0.39)
eGFRcr-cys	#	75.00 (61.32–85.95)	75.23 (61.66–86.08)	75.00 (61.32–85.95)	0.68 (0.71)
HMSE score	24.00 (19.00–27.00)	24.00 (19.00–27.00)	24.00 (19.00–27.00)	24.00 (19.00–27.00)	0.57 (0.75)


	Percentage prevalence				Chi-squared statistic (*P* value)
Sex (male)	46.12%	47.16%	46.97%	47.16%	0.03 (0.99)
Obesity (BMI ≥25 or Waist > 94 cm/80 cm) (%)	54.06%	53.74%	55.40%	53.74%	1.9 (0.39)
Hypertension (%)	43.31%	45.03%	45.51%	45.03%	0.17 (0.92)
(LDL > 100) (%)	59.73%	59.32%	60.04%	59.32%	0.39 (0.82)
Smoking (ever smoker) (%)	14.99%	16.58%	16.19%	16.58%	0.2 (0.90)
Alcohol consumption (Ever Drinker) (%)	14.83%	16.14%	15.68%	16.14%	0.29 (0.86)
Diabetes (%)	18.21%	24.63%	25.58%	24.63%	0.89 (0.64)
CVS event history (%)	5.58%	6.20%	6.28%	6.20%	0.02 (0.99)

BMI, body mass index; CVS, cardiovascular system; eGFRcr, estimated glomerular filtration rate-creatinine; eGFRcys, estimated glomerular filtration rate-cystatin C; eGFRcr-cys, estimated glomerular filtration rate-creatinine to cystatin C; HMSE, hindi mental state examination; LDL, low density lipoprotein.

# eGFR data was not available for all samples (*N* = 4096).

**Table 2:** This table depicts the distribution of eGFRcys, eGFRcr, and eGFRcr-cys values in all subjects with eGFRcys, eGFRcr, and eGFRcr-cys data respectively

eGFRcys (ml/min per 1.73 m^2^)	No. of subjects	%
≥ 90	205	7.87%
60–89	1124	43.16%
45–59	712	27.34%
30–44	397	15.25%
15–29	142	5.45%
< 15	24	0.92%
Total no. of subjects	2604	


eGFRcr (ml/min per 1.73 m^2^)	No. of subjects	%
≥ 90	1428	49.95%
60–89	1173	41.03%
45–59	169	5.91%
30–44	80	2.8%
15–29	9	0.31%
< 15	0	0.00%
Total no. of subjects	2859	


eGFRcr-cys (ml/min per 1.73 m^2^)	No. of subjects	%
≥ 90	472	18.13%
60–89	1542	59.22%
45–59	376	14.44%
30–44	141	5.41%
15–29	61	2.34%
< 15	12	0.46%
Total no. of subjects	2604	

eGFRcr, estimated glomerular filtration rate creatinine; eGFRcys, estimated glomerular filtration rate cystatin C.

### eGFR Variation With Geography

**Figure 1:**
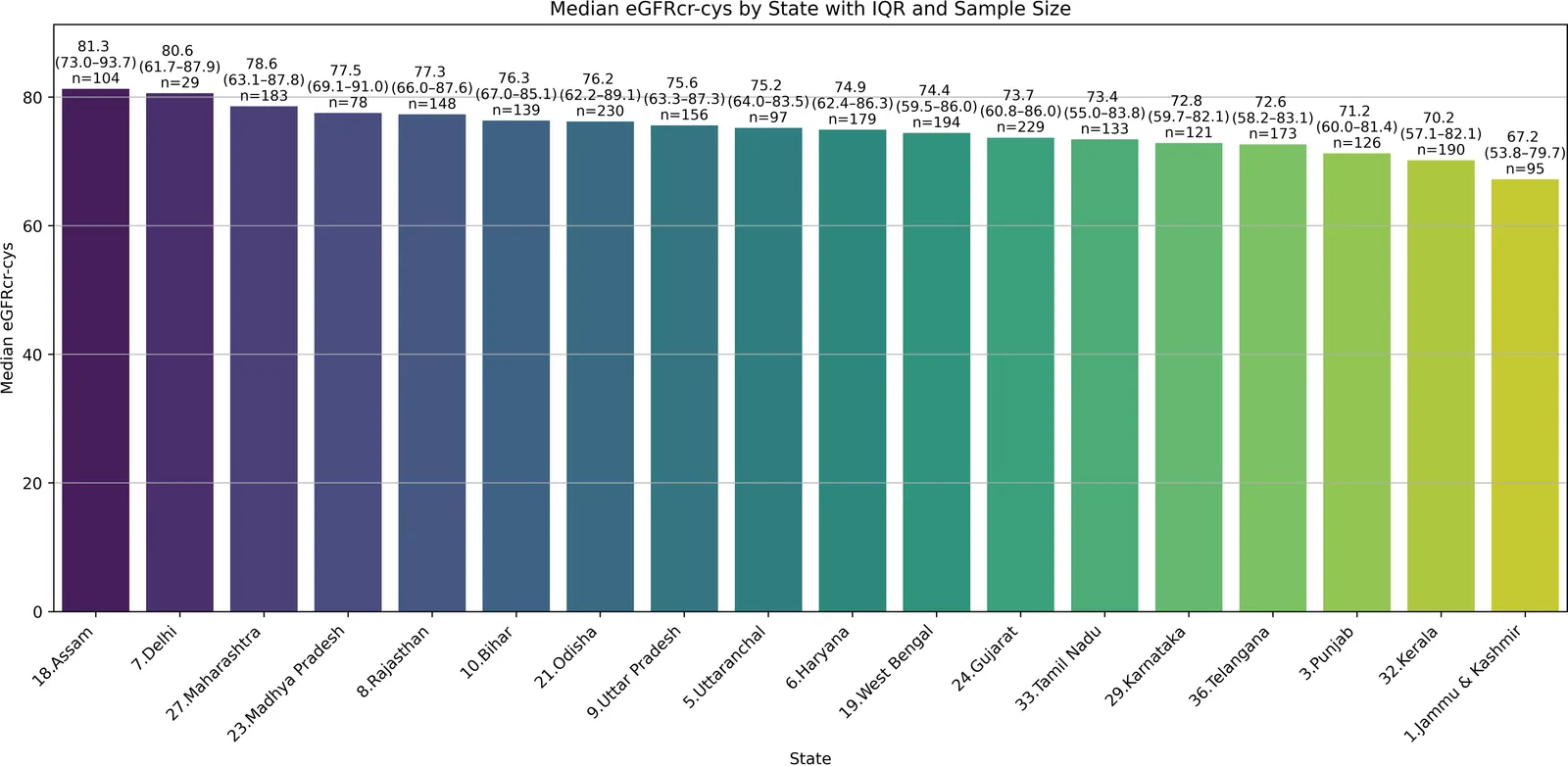
Depicts the state wise distribution of eGFR creatinine to cystatin C in the 18 states and union territories. eGFR, estimated glomerular filtration rate.

The prevalence of an eGFR less than 60 ml/min per 1.73 m^2^ was significantly higher when calculated using the combined formula (22.66%) compared with using creatinine alone (9.02%), with a statistically significant difference (*P* value < 0.001). As depicted in Figure 1, the state-wise distribution of eGFRcr-cys showed significant variation across the 18 states and union territories. The highest median eGFRcr-cys was observed in Assam (81.3 [70.3–93.7] ml/min per 1.73 m^2^), whereas the lowest was in Jammu and Kashmir (67.2 [53.8–79.7] ml/min per 1.73 m^2^).

### Correlation of Creatinine to Cystatin C Ratio With Cognitive Measures

Creatinine to cystatin C ratio was found to have a modest positive correlation with HMSE score (ρ = 0.18, *P* value < 0.001) and domain-wise cognitive scores as follows: orientation (ρ = 0.21, *P* value < 0.001), executive functioning (ρ = 0.22, *P* value < 0.001), language/fluency (ρ = 0.1, *P* value < 0.001), memory (ρ = 0.17, *P* value < 0.001), and visuospatial domain (ρ = 0.26, *P* value < 0.001).

### Logistic Regression

To predict the risk of cognitive impairment associated with impaired eGFR, logistic regression was performed. In unadjusted models, individuals with eGFR < 60 ml/min per 1.73 m^2^ had significantly higher risk of cognitive impairment when compared with those with eGFR ≥ 60 ml/min per 1.73 m^2^. However, this relationship was attenuated and no longer statistically significant after adjustment with age and sex in Model 2 and further adjustment with education and comorbidities in Model 3 (Table 3).

**Table 3:** Logistic regression analysis of the association between reduced kidney function (eGFR < 60 ml/min per 1.73 m^2^) and cognitive impairment. Odds Ratios represent the odds of cognitive impairment in participants with eGFR < 60 ml/min per 1.73 m^2^ compared with ≥ 60 ml/min per 1.73 m^2^

HMSE	eGFRcys	eGFRcr	eGFRcr-cys
	Odds ratio (95% CI), *P* value	Odds ratio (95% CI), *P* value	Odds ratio (95% CI), *P* value
Model 1^a^	1.161 (0.988–1.365), 0.070	1.156 (0.875–1.527), 0.309	1.106 (0.911–1.344), 0.309
Model 2^b^	0.963 (0.802–1.157), 0.688	0.962 (0.710–1.303), 0.801	0.913 (0.735–1.136), 0.415
Model 3^c^	1.100 (0.885–1.369), 0.390	1.419 (0.976–2.062), 0.067	1.082 (0.835–1.401), 0.551

eGFRcr, estimated glomerular filtration rate creatinine; eGFRcys, estimated glomerular filtration rate cystatin C.

a Model 1: unadjusted.

b Model 2: adjusted for age and sex.

c Model 3: adjusted for age, sex, years of education, obesity, LDL levels, diabetic status, smoking status, alcohol status, hypertension, CES-D score and previously diagnosed cardiovascular event history.

### Linear Regression

To further evaluate the relationship between kidney function and cognitive performance, linear regression was performed using eGFR as the continuous independent variable and cognitive test scores as continuous dependent variables. eGFR levels (eGFRcys, eGFRcr, and eGFRcr-cys) were significantly associated with scores in HMSE and domain-wise cognitive scores in unadjusted models. This relationship was attenuated and no longer statistically significant upon adjustment with age and sex in Model 2 and additionally with education and comorbidities in Model 3. (Table 4).

**Table 4:** Linear regression analysis showing the association between eGFR (per 10 ml/min per 1.73 m^2^ increase) and HMSE total score and domain-specific cognitive scores. Beta coefficients represent the mean change in cognitive score per 10-unit increase in eGFR

Model	eGFRcys	eGFRcr	eGFRcr-cys
HMSE	β-value (95% CI), *P-*value	β-value (95% CI), *P-*value	β-value (95% CI), *P-*value
Model 1^a^	0.136 (0.031–0.241), 0.011	0.222 (0.093–0.351), 0.001	0.148 (0.037–0.259), 0.009
Model 2^b^	−0.051 (−0.157 to 0.055), 0.348	−0.117 (−0.247 to 0.014), 0.080	−0.063 (−0.174 to 0.049), 0.272
Model 3^c^	−0.004 (−0.095 to 0.088), 0.939	0.080 (−0.034 to 0.194), 0.171	0.025 (−0.072 to 0.122), 0.614
Orientation			
Model 1^a^	0.016 (0.000–0.032), 0.045	0.027 (0.008–0.047), 0.006	0.017 (0.001–0.034), 0.042
Model 2^b^	−0.005 (−0.020 to 0.011), 0.557	−0.019 (−0.038 to 0.000), 0.045	−0.008 (−0.024 to 0.009), 0.356
Model 3^c^	0.001 (−0.012 to 0.014), 0.874	0.008 (−0.008 to 0.024), 0.335	0.004 (−0.010 to 0.018), 0.572
Executive functioning			
Model 1^a^	0.030 (0.013–0.048), 0.001	0.028 (0.007–0.050), 0.010	0.027 (0.008–0.045), 0.005
Model 2^b^	0.003 (−0.014 to 0.021), 0.718	−0.030 (−0.051 to -0.008), 0.007	−0.006 (−0.024 to 0.013), 0.544
Model 3^c^	0.01 (−0.004 to 0.023), 0.156	0.007 (-0.010 to 0.024), 0.427	0.009 (-0.006 to 0.023), 0.242
Language/Fluency			
Model 1^a^	0.015 (0.000–0.030), 0.053	0.027 (0.004–0.041), 0.017	0.015 (-0.001 to 0.031), 0.070
Model 2^b^	−0.007 (−0.023 to 0.009), 0.407	−0.015 (−0.035 to 0.004), 0.131	−0.010 (−0.027 to 0.007), 0.267
Model 3^c^	0.000 (−0.015 to 0.015), 0.981	0.011 (−0.008 to 0.029), 0.254	0.003 (−0.013 to 0.019), 0.708
Memory			
Model 1^a^	0.044 (0.025–0.062), < 0.001	0.042 (0.019–0.064), < 0.001	0.039 (0.020–0.058), < 0.001
Model 2^b^	−0.004 (−0.023 to 0.015), 0.667	−0.032 (−0.055 to −0.008), 0.008	−0.013 (−0.032 to 0.007), 0.203
Model 3^c^	0.004 (−0.013 to 0.020), 0.668	0.003 (−0.017 to 0.024), 0.747	0.002 (−0.015 to 0.019), 0.822
Visuospatial			
Model 1^a^	0.045 (0.028–0.062), < 0.001	0.039 (0.018–0.060), < 0.001	0.042 (0.024–0.060), < 0.001
Model 2^b^	0.020 (0.003–0.037), 0.019	−0.016 (−0.037 to 0.005), 0.138	0.012 (−0.006 to 0.030), 0.175
Model 3^c^	0.021 (0.007–0.034), 0.004	0.008 (−0.010 to 0.025), 0.383	0.018 (0.003–0.032), 0.019

eGFRcr, estimated glomerular filtration rate creatinine; eGFRcys, estimated glomerular filtration rate cystatin C; eGFRcr-cys, estimated glomerular filtration rate creatinine to cystatin C; HMSE, hindi mental state examination.

a Model 1: unadjusted.

b Model 2: adjusted for age and sex.

c Model 3: adjusted for age, sex, years of education, obesity, LDL levels, diabetic status, smoking status, alcohol status, hypertension, CES-D score and previously diagnosed cardiovascular event history.

### Restricted Cubic Spline Analysis

**Figure 2:**
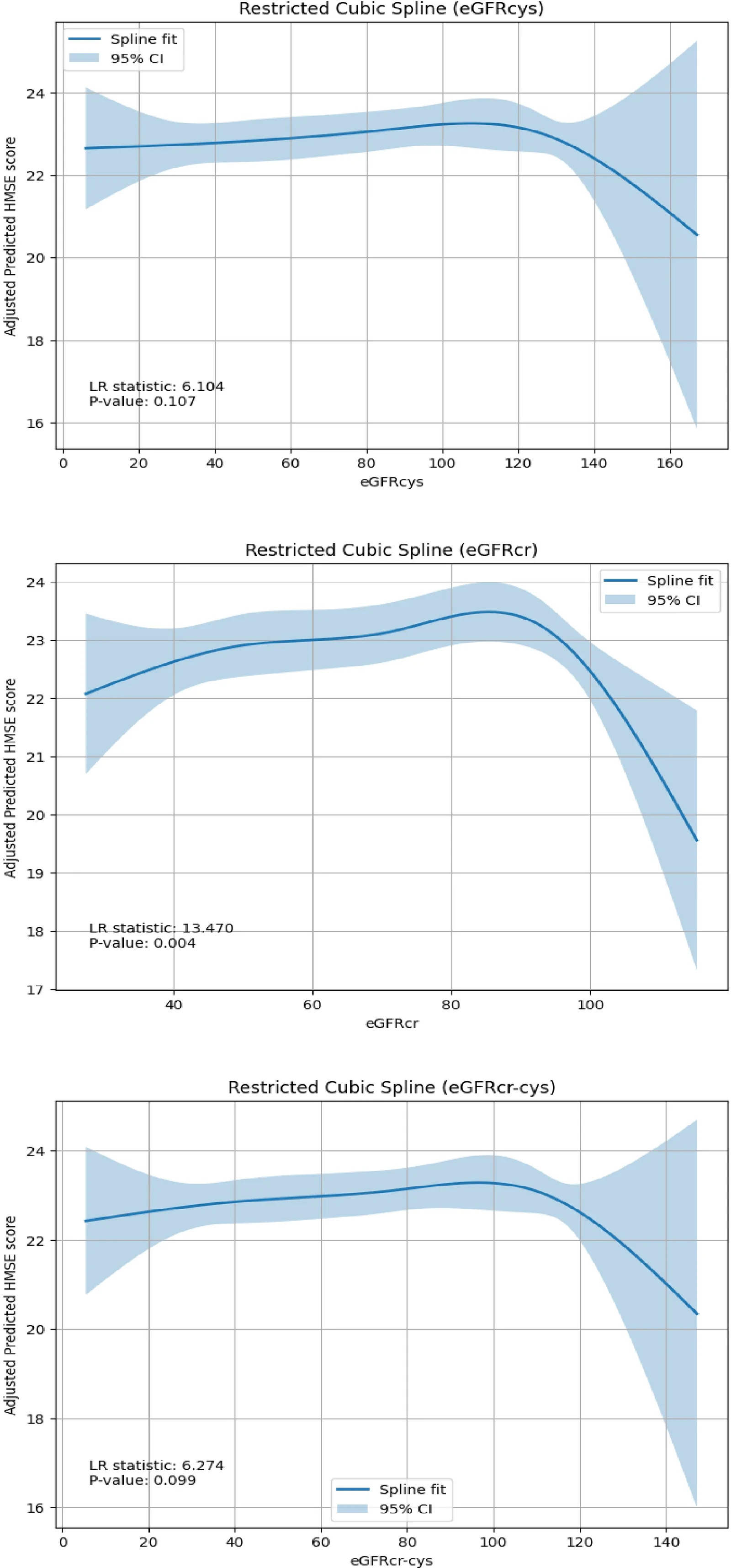
Restricted cubic spline showing the adjusted association between eGFR and HMSE score. Shaded areas indicate 95% confidence intervals. eGFR, estimated glomerular filtration rate

Restricted cubic spline analysis demonstrated a significant nonlinear association between eGFRcr and HMSE score (likelihood ratio statistic = 13.47, *P* value = 0.004). As shown in Figure 2, HMSE scores increased modestly with rising eGFRcr up to approximately 80 to 90 ml/min per 1.73m^2^, beyond which a decline was observed. Similar but nonsignificant trends were observed for eGFRcr-cys (*P* value = 0.099) and eGFRcys (*P* value = 0.107). Confidence intervals widened at extreme eGFR values, indicating greater uncertainty in these regions.

## Discussion

This study provides important insights into the association between kidney function and cognitive performance in community dwelling elderly in the pan-Indian context. We found a higher incidence of reduced eGFR in our study compared with those in other epidemiological studies and a null association between reduced kidney function and cognitive performance. However, we found a positive correlation between creatinine to cystatin C ratio (sarcopenia index) and HMSE, possibly indicating underlying mechanisms affecting both cognition and muscle mass in elderly.

A significant finding from our study is the remarkably high prevalence of eGFR < 60 ml/min per 1.73m^2^ in the elderly Indian population. We observed that this prevalence varied notably depending on the marker used—it was 48.96% with eGFRcys, 22.66% with eGFRcr-cys, and 9.02% with eGFRcr. These figures are substantially higher than those reported in previous cohort studies. For example, previous research on eGFRcys found prevalence rates (< 60 ml/min per 1.73m^2^) ranging from a low of 2.1% to a high of 16%^31^, ^32^, ^33^, ^34^, while eGFRcr prevalence was reported between 3.8% and 6.4%^34^^,^^35^, and eGFRcr-cys at 2.3%.^34^ The lower prevalence of reduced eGFR in previous studies of the Indian population can be partly attributed to the use of serum creatinine alone to calculate eGFR. Our data highlights this discrepancy; we found the prevalence of eGFRcr-cys < 60 ml/min per 1.73m^2^ to be 22.66%, which is more than double the 9.02% prevalence seen with eGFRcr alone (*P* value < 0.001).

This finding aligns with research showing a high discordance in South Asian populations, where eGFRcys is often more than 15% lower than eGFRcr.^36^^,^^37^ This discordance may be because of their distinct body composition and metabolic phenotype.^38^ These populations characteristically exhibit lower skeletal muscle mass and higher visceral adiposity at similar body mass index levels. Reduced muscle mass may lead to lower serum creatinine and consequent overestimation of kidney function when using eGFRcr. Conversely, increased visceral adiposity and chronic low-grade inflammation may elevate cystatin C levels independent of true GFR, resulting in lower eGFRcys. Therefore, individuals with discordantly lower eGFRcys may represent a subgroup with heightened metabolic and cardiovascular vulnerability that is not captured by creatinine alone. This has important implications for epidemiology of kidney dysfunction and risk stratification in South Asian aging cohorts.

The LASI-DAD cohort’s focus on cognitive impairment distinguishes it from the general elderly population. The resulting higher prevalence of comorbidities, like obesity and diabetes, may explain the increased rates of reduced eGFR observed in this group. In addition, other unknown population-specific differences in cystatin C^39^ and creatinine levels, lower nephron endowment, and exposure to environmental stressors such as recurrent dehydration (possibly exacerbated by global warming), environmental pollution, and the use of over-the-counter nephrotoxic agents like nonsteroidal anti-inflammatory drugs and pesticides because of its transmission in food chain, may all contribute to a decline in kidney function within this cohort.^40^, ^41^, ^42^ Our data also points to the existence of region-specific differences in prevalence of eGFR< 60 ml/min per 1.73 m^2^ within India, a finding that warrants further investigation.

Apart from these nuances, using cystatin C to estimate eGFR offers several other advantages, as follows: (i) less influence from muscle mass—It provides a more accurate indicator of kidney function, especially in the elderly where muscle mass varies^19^^,^^43^; (ii) higher sensitivity—eGFRcys is more sensitive in detecting early-stage kidney dysfunction compared with eGFRcr^44^; and (iii) better risk assessment—Cystatin C-based eGFR is superior for predicting risk of adverse health outcomes, even in obese individuals^45^^,^^46^ and is more effective at detecting advanced stages of renal disease than eGFRcr.^47^ Thus, a combined approach that incorporates both creatinine and cystatin C offers the best accuracy for clinical applications.^48^

To our knowledge, this is the first study to examine the relationship between creatinine based, cystatin C based, and combined eGFR measurements and cognitive performance in a large, community-dwelling older Indian adult cohort. We did not find any significant associations between eGFR and HMSE score and decreased eGFRcys, eGFRcr, and eGFRcr-cys (all < 60 ml/min per 1.73m^2^) did not show any elevated risk of cognitive impairment. In nonlinear analysis, the eGFRcr model reached statistical significance (*P* value = 0.004), suggesting that cognition varies across different eGFRcr levels in a nonlinear manner. However, the eGFRcys and eGFRcr-cys models were not significant. Because cystatin C-based and combined equations are more accurate and less influenced by muscle mass and frailty, the lack of replication across these equations weakens confidence that the finding reflects a true biological relationship. As creatinine is strongly influenced by muscle mass, the significant association may partly reflect the non-GFR determinants of creatinine rather than kidney function itself. The decline in HMSE scores at higher eGFR levels may reflect glomerular hyperfiltration, a marker of endothelial dysfunction that could contribute to cognitive impairment through cerebral microvascular injury.^49^^,^^50^

Our null result contrasts with some reports from Western cohorts but aligns with others. For example, Grantham *et al.* (ADNI cohort) found no association between eGFR and cognitive decline in older adults with only mild CKD.^51^^,^^52^ On the other hand, studies that include people with more severe CKD report cognitive effects, especially when advanced CKD is considered.^53^, ^54^, ^55^, ^56^, ^57^, ^58^ Indeed, newer evidence suggests that more advanced CKD stages are associated with cognitive impairment, whereas mild CKD shows little effect. In our sample, only about 8% of participants had an eGFRcr-cys < 45 ml/min per 1.73m^2^, so the cohort predominantly represents mild kidney dysfunction. Thus, the null association we observe likely applies to the lower end of the CKD spectrum (mild-to-moderate CKD), and we cannot rule out effects in more severe cases.

Previous research suggests that socioeconomic and educational variables (e.g., illiteracy and poverty) have a very strong impact on cognitive health in older Indians.^59^ It is conceivable that these socioeconomic determinants overshadow or mask any modest effect of kidney function on cognition in our sample. Additionally, differences in comorbidity profiles (e.g., rates of diabetes, hypertension, malnutrition etc.) or in the etiology of kidney dysfunction might modify the kidney–brain relationship in LMICs. Environmental factors (e.g., pollutants, infections) or genetic factors could also differ. Although the exact reasons remain speculative, it is plausible that findings from Western cohorts do not directly apply to older Indians. Future cross-cultural comparisons or pooled LMIC data would be valuable to test this hypothesis.

Our data reveals a modest positive correlation between serum creatinine levels, the creatinine to cystatin C ratio, and both HMSE and domain-specific cognitive scores. This aligns with existing research suggesting that an individual’s nutritional status, muscle mass, and sarcopenia are crucial determinants of cognitive performance.^24^, ^25^, ^26^ However, this relationship is likely bidirectional, with both reflecting a shared underlying process or declining cognition contributing to reduced mobility and subsequent sarcopenia, warranting further investigation into these mechanisms.

There are several limitations to our study. First, chronicity of kidney disease could not be determined as repeat measures of eGFR were not available. Second, 36.4 % of eGFR data was missing for LASI-DAD Wave 1 participants and this could lead to biased results from our analysis. Third, as LASI-DAD oversampled for participants with cognitive impairment, our population has a higher prevalence of comorbidities and therefore, might be different from the general population. Fourth, being a cross-sectional study, no causal relationship between kidney dysfunction and cognitive decline could be inferred from the findings. Fifth, although the cohort included subjects from 18 different states and union territories of India, the state-wise sample sizes were small and several states were not represented in this study. Lastly, data on hydration status, medication history, and family history of dementia and kidney disease, and genetic factors were not available.

In summary, kidney dysfunction was found to have a null association with cognitive impairment in this cohort. With a high burden of reduced eGFR and several risk factors of dementia, it is very important for the Indian population to be screened for implementation of early measures to prevent kidney dysfunction and dementia. Studies with larger sample size including more advanced stages of kidney dysfunction are required to further evaluate any potential association between kidney dysfunction and cognition. Addressing these challenges would help reduce the dual burden of CKD and cognitive impairment in elderly in LMICs.

## Disclosure

All the authors declared no competing interests.
